# Age-Related Tortuosity of Carotid and Vertebral Arteries: Quantitative Evaluation With MR Angiography

**DOI:** 10.3389/fneur.2022.858805

**Published:** 2022-04-29

**Authors:** Zhe Sun, Dengrong Jiang, Peiying Liu, Marco Muccio, Chenyang Li, Yan Cao, Thomas M. Wisniewski, Hanzhang Lu, Yulin Ge

**Affiliations:** ^1^Department of Radiology, NYU Grossman School of Medicine, New York, NY, United States; ^2^Vilcek Institute of Biomedical Science, NYU Grossman School of Medicine, New York, NY, United States; ^3^Department of Radiology and Radiological Science, Johns Hopkins University School of Medicine, Baltimore, MD, United States; ^4^Department of Mathematical Sciences, University of Texas at Dallas, Richardson, TX, United States; ^5^Department of Neurology, NYU Grossman School of Medicine, New York, NY, United States; ^6^Department of Pathology, NYU Grossman School of Medicine, New York, NY, United States; ^7^Department of Psychiatry, NYU Grossman School of Medicine, New York, NY, United States; ^8^Center for Cognitive Neurology, NYU Grossman School of Medicine, New York, NY, United States

**Keywords:** aging, carotid artery, vascular tortuosity, blood flow, magnetic resonance angiography

## Abstract

**Background and Purpose:**

The vascular tortuosity (VT) of the internal carotid artery (ICA), and vertebral artery (VA) can impact blood flow and neuronal function. However, few studies involved quantitative investigation of VT based on magnetic resonance imaging (MRI). The main purpose of our study was to evaluate the age and gender effects on ICA and VA regarding the tortuosity and flow changes by applying automatic vessel segmentation, centerline tracking, and phase mapping on MR angiography.

**Methods:**

A total of 247 subjects (86 males and 161 females) without neurological diseases participated in this study. All subjects obtained T1-weighted MRI, 3D time-of-flight MR angiography, and 2D phase-contrast (PC) MRI scans. To generate quantitative tortuosity metrics from TOF images, the vessel segmentation and centerline tracking were implemented based on Otsu thresholding and fast marching algorithms, respectively. Blood flow and velocity were measured using PC MRI. Among the 247 subjects, 144 subjects (≤ 60 years, 49 males/95 females) were categorized as the young group; 103 subjects (>60 years, 37 males/66 females) were categorized as the old group.

**Results:**

Independent *t*-test showed that older subjects had higher tortuosity metrics, whereas lower blood flow and velocity than young subjects (*p* < 0.0025, Bonferroni-corrected). Cerebral blood flow calculated using the sum flux of four target arteries normalized by the brain mass also showed significantly lower values in older subjects (*p* < 0.001). The age was observed to be positively correlated with the VT metrics. Compared to the males, the females demonstrated higher geometric indices within VAs as well as faster age-related vascular profile changes. After adjusting age and gender as covariates, maximum blood velocity is negatively correlated with geometric measurements. No association was observed between blood flux and geometric measures.

**Conclusions:**

Vascular auto-segmentation, centerline tracking, and phase mapping provide promising quantitative assessments of tortuosity and its effects on blood flow. The neck arteries demonstrate quantifiable and significant age-related morphological and hemodynamic alterations. Moreover, females showed more distinct vascular changes with age. Our work is built upon a comprehensive quantitative investigation of a large cohort of populations covering adult lifespan using MRI, the results can serve as reference ranges of each decade in the general population.

## Introduction

The internal carotid artery (ICA) and vertebral artery (VA) are main feeding arteries of the brain and play critical roles in supplying energy and maintaining normal neuronal function. It has been widely reported in the literatures that extracranial arteries are prone to demonstrate geometric and morphological variants with normal aging (1–3). Due to the long courses of extracranial arteries in the neck region, the age-related morphological variations represent distinctly as abnormal twisting, turns, and loops found in the elderly in contrast to the normal anatomical turns. These vascular morphological changes can limit the blood flow, which may lead to stroke and other ischemic events, such as the transit ischemic attack and vertigo (4–8). Thus, it is clinically important to assess vascular tortuosity (VT) for a better understanding how VT can affect the blood flow. Non-invasive clinical imaging techniques, particularly ultrasound and MRI, stand at the epicenter of VT and flow assessment in the elderly population. Compared to ultrasound, MRI is operator-independent with higher fidelity, larger field of view (FOV), and greater capacity of deep vessel detection (e.g., VA). While these age-related tortuosity changes raise the awareness of their etiology, pathophysiological mechanisms, and clinical monitoring, few published studies have quantified the VT and its correlation with blood flow using MRI.

The aging and the mechanical injury of vessel wall were proposed as the two important pathological processes of VT. With aging, degenerative changes of extracellular matrix and endothelium result in arterial wall remodeling, stiffness, and tortuosity (9). The mechanical factors also increase the incidence of VT secondary to the repetitive cardiac pulsatile flow effects accumulated over the lifespan in the elderly. Responding to constantly imposed forces of blood flow, the vessels become tortuous associated with elastin degradation and proinflammatory signaling activation (10, 11), Conversely, VT impedes flow effects and affect vascular wall shear stress (WSS) associated with platelets activation and thrombosis (12). These have been observed clinically with decreased cerebral blood flow (CBF) in the elderly with more tortuosity vessels (13, 14). Ultrasound studies have shown that blood velocities in ICAs were stable up to the age of 40–45 years and decreased afterward associated with increased tortuosity appearances (15, 16). However, the clear associations between tortuosity and flow changes in normal aging have not been established.

Time-of-flight (TOF) and phase-contrast (PC) imaging are two MR angiographic (MRA) techniques commonly used in the routine clinical settings for vascular characteristic inspection (17–20). The present study aims to comprehensively quantify the age-related VT of ICAs, and VAs and their blood flow by applying automated vessel segmentation, centerline tracking, and phase mapping on MRA. The methods developed in this study can potentially be implemented in clinical scans for grading the vessel integrity of major brain feeding arteries by referring to quantitative metrics of vascular characteristics.

## Materials and Methods

### Study Population

After the institutional review board's approval, 251 subjects were initially recruited, and four of them were excluded due to the abnormal arteries on TOF–MRA or missing unilateral VA, which is a type of variant anatomy. Finally, 247 individuals (86 males and 161 females) were enrolled with age ranging from 20 to 88 years in this retrospective study. Among the 247 subjects, 144 participants younger than and including 60 years old were classified as the young group; 103 participants older than 60 years old were classified as the old group. The participants of our study were subset of the Dallas Lifespan Brain Study (DLBS) designed to understand the preservation and decline of cognitive function. All participants underwent screening and had no contraindications to MRI scan (pacemaker, implanted metallic objects, and claustrophobia) and were generally of good health, without serious medical conditions such as neurological disease, brain injury, shaking, or medications affecting cognitive functions (7). All participants were right-handed native English speaker with Mini-Mental State Exam (MMSE) score of 26 or greater. Among the 247 participants, 232 had blood pressure (BP) (i.e., systolic and diastolic) that was measured 5 times around the time of MRI scan. The mean systolic and diastolic BP of the five measurements was then used for the further analyses related to tortuosity (21). Among them, 33 participants with a diagnosis of hypertension were taking antihypertensive medications, most of which were angiotensin covering enzyme inhibitors, beta-blockers, and angiotensin II receptor antagonists (7).

### The MR Imaging Acquisition

The MR imaging was performed using a 3T MRI system with an 8-channel head coil. Also, T1-weighted magnetization-prepared-rapid-acquisition-of-gradient-echo (MPRAGE), 3D-TOF, and 2D non-gated PC–MRI scans were acquired for each subject. The MPRAGE sequence used the following imaging parameters: Repetition time (TR)/echo time (TE)/flip angle (FA) = 8.1 ms/3.7 ms/12°, voxel size = 1 mm × 1 mm × 1 mm. For the purpose of the vessel segmentation and PC–MRI positioning, the TOF was acquired with the following parameters: TR/TE/FA = 23 ms/3.45 ms/18°, voxel size = 1.0 mm × 1.0 mm × 1.5 mm, number of slices = 47, a 60-mm saturation band positioned above the imaging slab, scan time = 1 min 26 s. The top of the angiographic imaging slab was positioned at the level of the bottom of pons, with the bottom FOV margin at the level around cervical spine C4, covering the cervical segment of ICA. Based on the coronal maximum intensity projection (MIP) images from TOF, the non-gated PC–MRI scan covering the four feeding arteries was conducted around the level of cervical spine C3 above the carotid bifurcations with following parameters: TR/TE/FA = 20 ms/7 ms/15°, voxel size = 0.45 mm × 0.45 mm × 5 mm, velocity encoding (VENC) = 80 cm/s, scan duration = 30 s.

### Segmentation, Centerline Tracking and Quantitative Tortuosity Measurements of Neck Vessels

**Figure 1:**
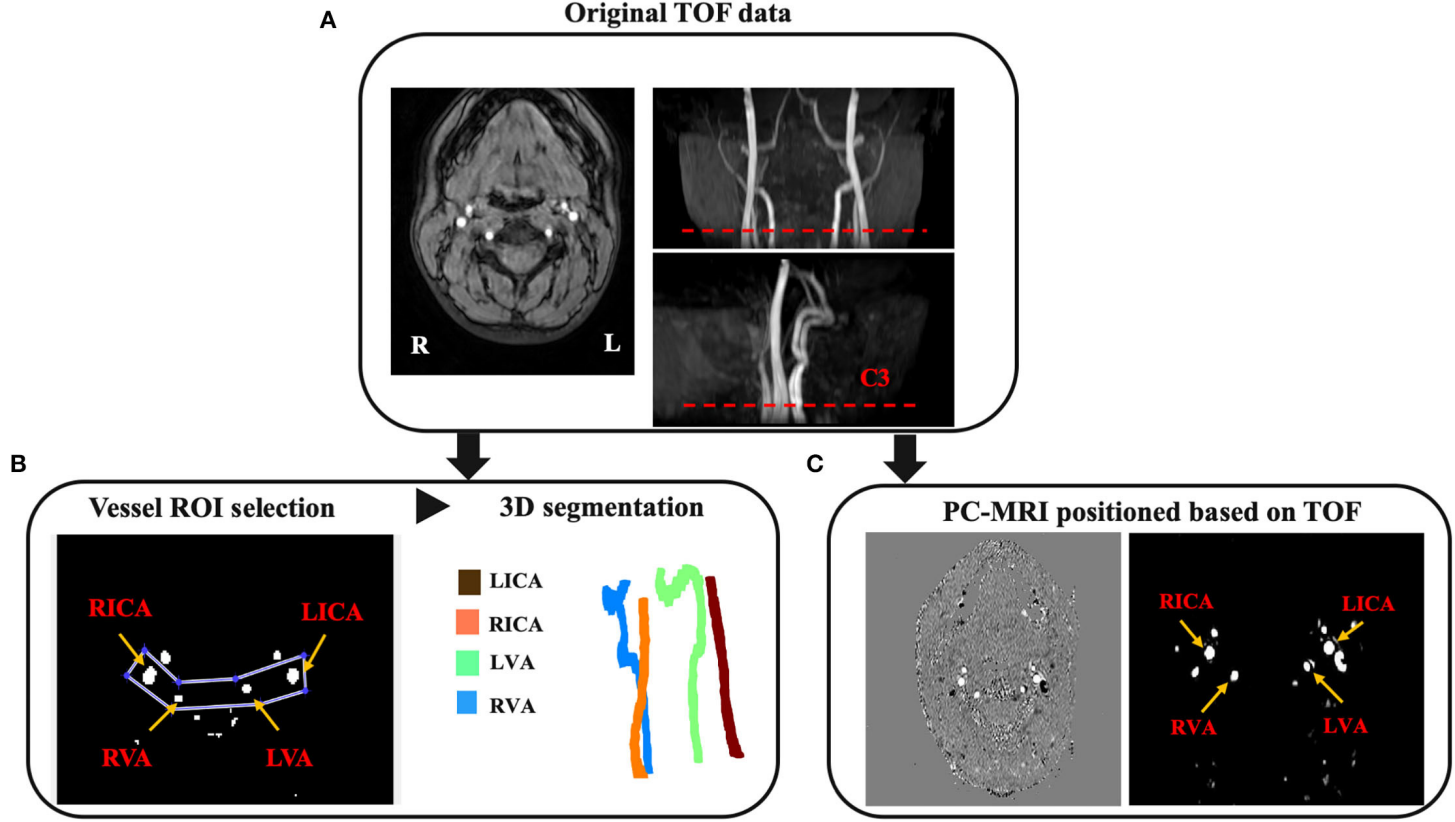
Image postprocessing workflow. **(A)** The TOF magnetic resonance angiography (MRA) including raw axial images, coronal and sagittal MIP images are acquired for the purpose of vessel segmentation and PC-MRI positioning. The PC-MRI scan plane is positioned around the level of cervical vertebrate C3 (red dash line), where above the carotid bifurcations. **(B)** The ROI enclosing target arteries and surrounding tissues are selected for the purpose of vessel segmentation and tortuosity measurement. Arteries can be detected based on the mean and standard deviation of the object region. **(C)** Corresponding phase image (left) and magnitude image (right) from the feeding arteries positioned at the level around C3 [red dash line in **(A)**].

We segmented and tracked the centerline of the vessels from 3D-TOF angiogram using the in-house code run on MATLAB 2020a (MATLAB and statistics Toolbox Release 2020a, MathWorks, Inc. Natick, MA). Figure 1 shows the image postprocessing workflow. A preliminary ROI enclosing the targeting arteries was drawn. The artery region *T*(*x*) consisting pixels *x* was determined using the following equation:


$$\begin{array}{l} T\left(x\right)\;=\;I\left(x\right)-\mu -j\cdot \;\sigma , \end{array}$$

where *I*(*x*) is the intensity of the object region (Ω) consisting of both the arteries and the surrounding tissues; μ and σ are the mean and standard deviation intensities of Ω, respectively. Since the signal intensities of the arteries are inhomogeneous, we need to start with a high value of *j* and decrease it gradually until an optimal value was reached. The optimal value of *j*, which varied between subjects, should be high enough to exclude surrounding tissue meanwhile low enough to include the arteries (22). Although *j* was determined subject-by-subject basis, we found an empirical search range of 6–4 in descending order was applicable for most cases. After the artery pixels were determined for each slice, they were connected if their faces or edges touched so as to get the 3D segmentation of arteries.

**Figure 2:**
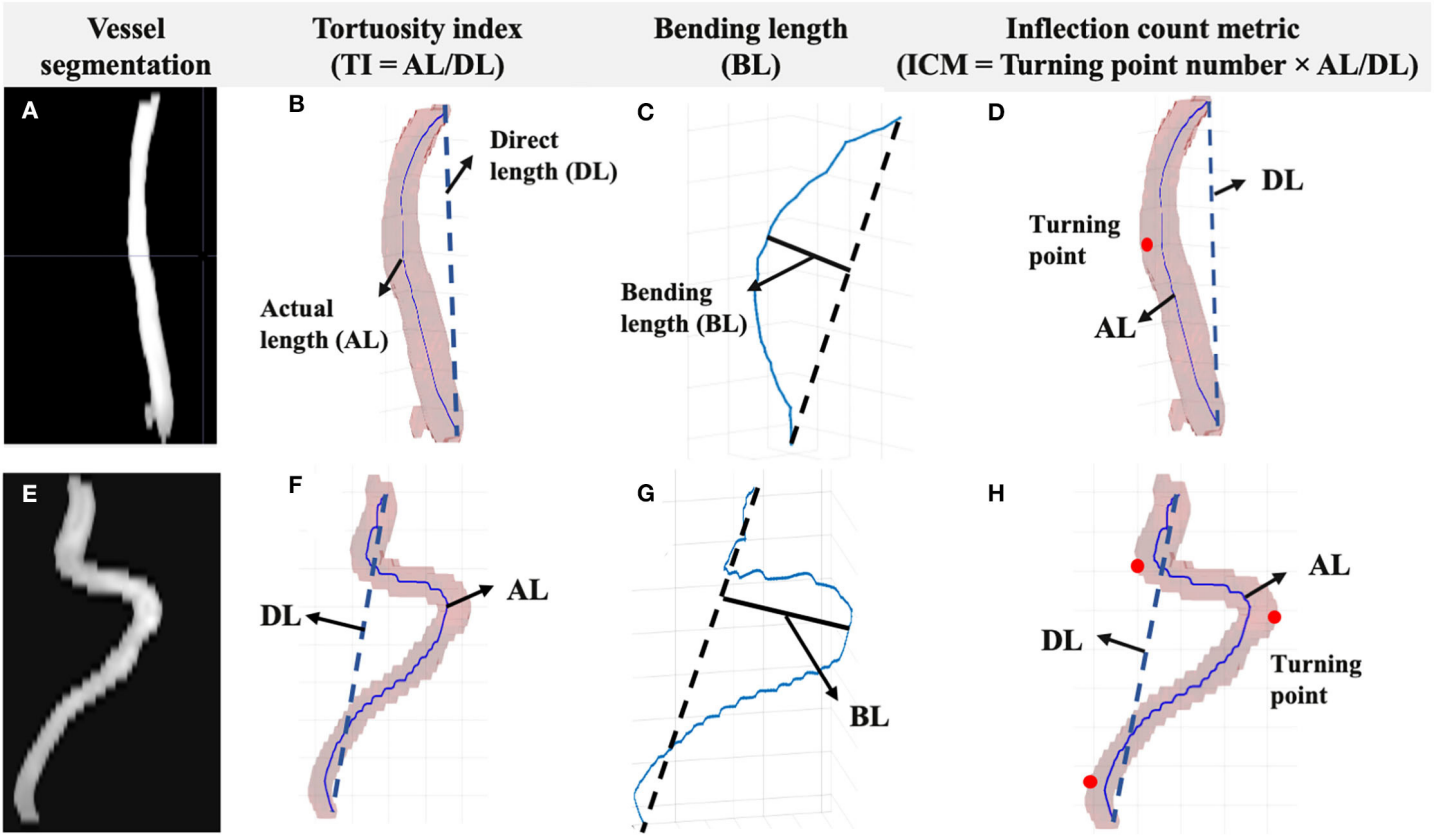
Segmentation and the centerline of the vessel were acquired. The measurements included TI, BL, and ICM. **(A)** Left ICA vessel segmentation of a young subject (≤ 60 years old). **(B)** The TI is the ratio between AL and DL. **(C)** The BL is the distance between the foremost points on the vessel path and the start-end points link. **(D)** The ICM is the product of turning points number (red dots) and TI. Corresponding vessel segmentation and measurements of an old subject (>60 years old) are shown in parts **(E–H)**.

Based on the arterial surface derived from 3D segmentation, the arterial centerline was acquired using multi-stencils fast marching algorithm, which calculates the shortest distance from a source point to all other pixels in an image volume by solving the Eikonal equation along stencils that cover neighboring points entirely (23). The centers of these level curves formed the skeleton representing the artery. The coordinates of the extracted arterial skeleton can be acquired to quantitatively assess the VT. We proposed to use tortuosity index (TI), bending length (BL), and inflection count metric (ICM) as quantitative VT indices (Figure 2). The TI is the ratio between the actual length (AL) and the direct length (DL). The BL is the maximum perpendicular distance between the AL and the DL, reflecting the extent of curving. The ICM is the product of turning points number (*N*) and TI, which takes turning frequency into consideration. Here, *N* was determined by visually checking the centerline skeleton.

### Postprocessing of PC-MRI

Each PC–MRI scan generated a phase image and a magnitude image. The data processing of PC–MRI followed previously reported method (24). Briefly, the region of interests (ROIs) were manually drawn on the magnitude images by circling the targeted artery (bilateral ICAs and VAs) based on the brightness of the voxels (25). A signal intensity threshold set to be 5 times the background noise was then applied to the magnitude image to get vessel mask. The mask was then applied to the phase image to calculate blood flux and maximum blood velocity (MaxV). The unit volume CBF (in ml/min/100 g) was calculated as the sum of blood flux of the four arteries normalized to the brain's parenchyma mass obtained from T1WI.

### Evaluation of Accuracy and Reproducibility

We used maximal intensity projection (MIP) images (both coronal and sagittal planes) generated from TOF–MR angiogram as a reference to evaluate the accuracy of arterial vessel segmentation. After overlaying the acquired centerline on the arterial segment, it would be easy to identify false branches and tell the tracking accuracy. All the segmented arteries and centerline images were visually inspected and the arteries with incomplete processing or aborted algorithms were excluded.

In addition, a scan–rescan study was performed to evaluate the reproducibility of 3D-TOF vessel tortuosity measurements. Seven young, healthy subjects (4 males, 3 females, 26.4 ± 3.7 years) underwent two MRI sessions with a 5-min break in between. During each session, 3D-TOF angiogram and PC-MRI scan were acquired. Inter-session coefficient of variation (CoV) of tortuosity measurements was calculated as follows:


$$\begin{array}{l} CoV_{intersession}\;=\;\frac{\left|measument_{1}\;-\;measurement_{2}\right|}{\sqrt{2}\cdot Mean\left(measurement_{1},measurement_{2}\right)\;} \end{array}$$

where *measurement*_1_ and *measurement*_2_ represent the TI, BL, or ICM from two scan and rescan sessions, respectively. These two separate scans are supposed to consist of repositioning error, and TOF–MRA noise.

### Statistical Analysis

Two-tailed *t*-test was applied to identify VT and flow differences between young and old groups. Since 20 analyses on the same dependent variable were being performed, Bonferroni-corrected alpha (α_*corrected*_ = 0.0025) was calculated to control the Type I error and a value of *p* ≤ 0.0025 was considered statistically significant for multiple comparisons and correlation studies. The Pearson correlation coefficients (*r*) were calculated to reveal the relationship between age and each vascular feature respectively. The multivariate linear regression was also performed using age as independent variable, VT and flow measurements as dependent variables, and BP (mean systolic and diastolic) measurements as covariates. Since the previous study showed hypertension might be associated with arteriosclerotic and vessel wall changes, we included these analyses to remove the hypertension effects from aging. To determine whether there is a statistically significant difference of VT and flow measurements between hypertension and normotension, 33 age/gender-matched non-hypertensive subjects (age: 67.91 ± 11.85; 24 females/9 males) were chosen to compare with the 33 hypertensive subjects (age: 69.53 ± 12.4; 24 females/9 males) using Student's *t*-test. We further split the cohort into females and males. Two-tailed *t*-test was applied to identify VT and flow differences between male and female groups. Univariate linear regression analyses were performed to investigate relationship and coefficients between age and vascular features for different genders. The association between tortuosity and blood flow was calculated using multivariate linear regression, treating age, gender, and each VT measurement as regressors. GraphPad Prism 8 was used for the statistical analysis.

## Results

### Demographic Characteristics

The descriptive statistics associated with blood pressure, and vascular profiles of ICA across the seven age decade groups are reported in Table 1. A detailed information of females and males in each decade group are reported in Supplementary Tables 1 and 2, respectively. Among 247 subjects (aged 53.23 ± 19.62 years, 86 males/161 females), we identified 144 younger subjects with age less than and including 60 years (aged 39.20 ± 12.11, 49 males/95 females) and 103 older subjects with age more than 60 years (aged 72.86 ± 7.5, 37 males/66 females). The mean systolic (137.8 ± 16.5 mm Hg) and diastolic (83.2 ± 9.6 mm Hg) BP of participants in old group was significantly higher than that of young subjects (116.7 ± 13.6 and 78.7 ± 9.6, respectively) (*p* < 0.001). Chi-square analysis did not reveal a statistically significant difference in gender for each age group.

**Table 1:** The ICA VT and flow measurements of each age decade.

**Age group**		**20–30**	**31–40**	**41–50**	**51–60**	**61–70**	**71–80**	**>80**
		**(*n* = 42)**	**(*n* = 32)**	**(*n* = 36)**	**(*n* = 34)**	**(*n* = 46)**	**(*n* = 32)**	**(*n* = 25)**
**Demographic data**								
Age		24.31 ± 2.97	34.88 ± 2.73	45.58 ± 3.02	54.91 ± 3.03	66.24 ± 3.16	73.4 ± 2.60	83.67 ± 2.46
Gender		14 M/ 28 F	10 M/ 22 F	15 M/ 21 F	10 M/ 24 F	17 M/ 29 F	10 M/ 22 F	10 M/ 15 F
Hypertension		0	1	2	4	11	9	7
BP		111/75	111/77	119/80	127/83	136/84	139/84	141/81
**The VT measurements**								
TI	LICA	1.70 ± 0.37	1.72 ± 0.30	1.83 ± 0.43	2.09 ± 0.70	2.36 ± 0.69	2.59 ± 0.76	2.87 ± 1.04
	RICA	1.74 ± 0.43	1.89 ± 0.53	1.92 ± 0.53	2.22 ± 0.61	2.64 ± 0.98	2.57 ± 0.61	3.00 ± 0.79
BL	LICA	19.84 ± 7.37	21.77 ± 6.71	22.69 ± 7.01	28.44 ± 10.4	31.44 ± 10.45	36.26 ± 12.97	39.24 ± 13.48
	RICA	19.49± 7.44	24.54 ± 8.05	24.30 ± 6.37	31.1 ± 10.45	34.56 ± 12.93	35.05 ± 8.51	43.05 ± 10.6
ICM	LICA	2.37 ± 1.22	2.38 ± 1.01	3.18 ± 2.22	4.53 ± 2.69	5.25 ± 2.49	5.70 ± 2.74	6.22 ± 3.78
	RICA	2.73± 2.12	3.17± 1.87	3.51 ± 2.56	4.8 ± 2.79	6.02 ± 3.98	6.14 ± 3.03	6.68 ± 4.11
**Flow measurements**								
Blood flux	LICA	286.37 ± 59.09	269.90 ± 46.10	246.26 ± 51.93	245.42 ± 56.47	222.71 ± 56.12	196.01 ± 62.71	191.82 ± 54.8
	RICA	275.43 ± 56.67	265.6 ± 49.56	258.88 ± 51.98	222.88 ± 56.54	213.47 ± 59.95	200.86 ± 52.16	199.06 ± 45.4
MaxV	LICA	36.17 ± 8.20	34.69 ± 9.33	34.7 ± 8.31	31.26 ± 8.4	25.93 ± 8.05	25.54 ± 6.88	22.07 ± 6.0
	RICA	36.61 ± 8.18	36.18 ± 8.04	37.22 ± 8.81	32.21 ± 9.42	26.81 ± 9.1	26.18 ± 5.86	25.19 ± 6.58

*BP, blood pressure presenting as systolic/diastolic with 15 subjects' measurements missing. LICA, left internal carotid artery; RICA, right carotid artery; TI, tortuosity index; BL, bending length (mm); ICM, inflection count metric; Blood flux in ml/min; MaxV, maximum blood velocity, in cm/s*.

### Group Comparison of VT and Blood Flow

**Figure 3:**
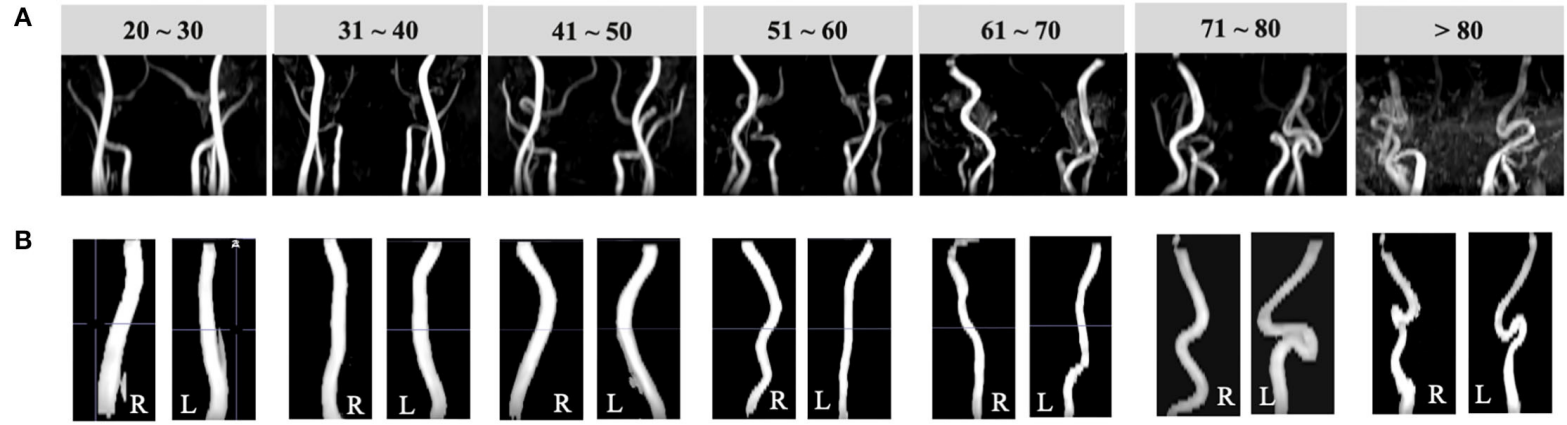
Illustrations of vessel segmentation for each decade over the adult lifespan. **(A)** Coronal TOF images (radiological view) of subjects from each decade demonstrate arteries become more tortuous with aging. **(B)** Left and right ICA segmentation results of subjects of age from 20 to 80 years.

As shown in Figure 3, VTs were widely observed in the older subject group. Quantitatively, ICAs of older subjects demonstrated significantly higher TI (left = 2.56 ± 0.83, right = 2.70 ± 0.84), BL (left = 34.78 ± 12.3, right = 36.92 ± 11.7), and ICM (left = 5.65 ± 2.92, right = 6.28 ± 3.76) than younger subjects' TI (left = 1.83 ± 0.49, right = 1.93 ± 0.55), BL (left = 22.96 ± 8.6, right = 24.49 ± 9.11), and ICM values (left = 3.09 ± 2.08, right = 3.52 ± 2.46) (*p* < 0.001 for all; details can be found in Table 1); significantly higher ICM of left vertebral artery (LVA) (*p* < 0.001) and BL of right vertebral artery (RVA) (*p* < 0.001) could be observed in older subjects. The TI, BL values of LVA and TI, ICM of RVA did not show significant difference. The old subjects demonstrated significantly lower blood flux, and MaxV for all arteries compared to young subjects. Compared to global-wise reports, unit mass CBF was significantly lower in old (49.15 ± 10.56 ml/min/100 g) as compared to young participants (55.77 ± 9.89 ml/min/100 g) (*p* < 0.001). A detailed descriptive statistics and *p*-values can be found in Table 2.

**Table 2:** Comparison of VT and flow measurements between young (*n* = 144) and old (*n* = 103) groups.

	**LICA**			**RICA**		
	**Young**	**Old**	***p***	**Young**	**Old**	***p***
**The VT measurements**						
TI	1.83 ± 0.49	2.56 ± 0.83	<0.001	1.93 ± 0.55	2.70 ± 0.84	<0.001
BL	22.96 ± 8.6	34.78 ± 12.33	<0.001	24.49 ± 9.11	36.92 ± 11.7	<0.001
ICM	3.09 ± 2.08	5.65 ± 2.92	<0.001	3.52 ± 2.46	6.28 ± 3.76	<0.001
**Flow measurements**						
Blood flux	263.01 ± 56.3	206.08 ± 59.1	<0.001	256.70 ± 56.7	206.05 ± 54.0	<0.001
MaxV	34.31 ± 8.6	24.91 ± 7.4	<0.001	35.63 ± 8.7	26.22 ± 7.6	<0.001
	**LVA**			**RVA**		
**The VT measurements**						
TI	1.24 ± 0.2	1.32 ± 0.26	0.01^*^	1.3 ± 0.4	1.36 ± 0.27	0.18^*^
BL	4.77 ± 2.52	5.83 ± 2.94	0.003^*^	5.07 ± 2.79	6.44 ± 3.18	<0.001
ICM	1.54 ± 0.76	2.14 ± 1.27	<0.001	1.78 ± 1.75	2.28 ± 1.29	0.01^*^
**Flow measurements**						
Blood flux	102.54 ± 43.3	84.38 ± 38.0	<0.001	86.83 ± 39.7	68.34 ± 32.8	<0.001
MaxV	22.99 ± 6.7	17.67 ± 5.3	<0.001	20.09 ± 6.0	15.75 ± 4.8	<0.001

*LICA, left internal carotid artery; RICA, right carotid artery; LVA, left vertebral artery; RVA, right vertebral artery; TI, tortuosity index; BL, bending length (mm); ICM, inflection count metric; Blood flux is in ml/min; MaxV, maximum blood velocity, is in cm/s. Adjusted p ≤ 0.0025 is the threshold for significance*.

* *There is no significant difference (p > 0.0025)*.

### Age Effects on VT and Flow Measurements

According to the Pearson correlation results, TI (*r* of left: 0.52 and right: 0.52), BL (*r* of left: 0.55 and right: 0.61), and ICM (*r* of left: 0.52 and right: 0.43) were positively correlated with age for bilateral ICAs (*p* < 0.001). The ICM of bilateral VAs (*r* of left: 0.29 and right: 0.19, *p* < 0.001 and *p* = 0.002, respectively) and BL of RVA (*r* = 0.2, *p* = 0.002) were positively correlated with age. Significantly, the negative correlations were identified between the flow measurements and age for all arteries (*p* < 0.001). A detailed *r* and *p-*values were summarized in Table 3. Separate multivariate linear regression was performed and showed statistical significance for tortuosity measurements of bilateral ICAs, as well as ICM of bilateral VAs in relation with age after adjusting mean systolic and diastolic BP as covariates (*p* < 0.0025 for all measurements). Similarly, the multivariate linear regression result showed significant associations for blood flux and maximum velocity of all four arteries (*p* < 0.001) in relation with age.

**Table 3:** Age in relation to the VT and blood flow measurements.

	**LICA**		**RICA**		**LVA**		**RVA**	
	***r***	***p***	***r***	***p***	***r***	***p***	***r***	***p***
**The VT measurements**	
TI	0.52	<0.001	0.52	<0.001	0.13	0.04^*^	0.07	0.26^*^
BL	0.55	<0.001	0.61	<0.001	0.15	0.02^*^	0.20	0.002
ICM	0.52	<0.001	0.43	<0.001	0.29	<0.001	0.19	0.002
**Flow measurements**	
Blood flux	−0.52	<0.001	−0.48	<0.001	−0.27	<0.001	−0.29	<0.001
MaxV	−0.52	<0.001	−0.49	<0.001	−0.44	<0.001	−0.41	<0.001

*r: Pearson correlation coefficient; Adjusted p ≤ 0.0025 is the threshold for significance*.

* *There is no significant difference (p > 0.0025)*.

### Hypertension Effects on VT and Flow Measurements

Student's *t*-test did not show statistically significant difference in VT and flow measurements for all vessels between hypertensive subjects and age- and gender-matched non-hypertensive subjects (*p* > 0.05 for all measurements). The multivariate linear regression showed systolic and diastolic BP measured before MRI did not significantly associated with VT and blood flow measurements (*p* > 0.05 for all measurements) after adjusting for age as a covariate.

### Gender Effects on VT and Flow Measurements

To further investigate the gender effects, we then compared VT and flow measurements between males (age: 53.62 ± 20.07) and females (age: 53.04 ± 19.45) regardless of age. The VT and the flow of bilateral ICAs did not show any significant difference (*p* > 0.0025) between males and females. However, within the VA system, females showed significantly higher values of RVA TI (right = 1.37 ± 0.39), BL (left = 5.67 ± 2.87, right = 6.30 ± 3.13), and ICM (left = 1.94 ± 1.18, right = 2.28 ± 1.84) than those of males (TI: right = 1.22 ± 0.21; BL: left = 4.34 ± 2.28, right = 4.43 ± 2.28; ICM: left = 1.50 ± 0.64, right: 1.44 ± 0.68) (*p* < 0.001). Among flow measurements, males had lower MaxV for bilateral VAs (*p* < 0.0025). A detailed descriptive statistics and *p-*values can be found in Table 4.

**Table 4:** Comparison of VT and flow measurements between males (*n* = 86) and females (*n* = 161) regardless of age.

	**LICA**			**RICA**		
	**Male**	**Female**	***p***	**Male**	**Female**	***p***
**The VT measurements**	
TI	2.08 ± 0.73	2.16 ± 0.76	0.46^*^	2.13 ± 0.76	2.31 ± 0.79	0.08^*^
BL	28.5 ± 12.12	27.57 ± 11.71	0.56^*^	29.67 ± 11.5	29.68 ± 12.2	0.99^*^
ICM	3.68 ± 2.87	4.41 ± 2.68	0.05^*^	3.99 ± 3.32	5.03 ± 3.35	0.02^*^
**Flow measurements**	
Blood flux	230.06 ± 60.3	244.18 ± 65.1	0.02^*^	223.55 ± 58.3	242 ± 61.6	0.02^*^
MaxV	28.62 ± 7.9	31.33 ± 9.95	0.02^*^	29.8 ± 8.39	32.72 ± 9.89	0.02^*^
	**LVA**			**RVA**		
**The VT measurements**						
TI	1.22 ± 0.21	1.30 ± 0.24	0.006^*^	1.23 ± 0.25	1.37 ± 0.39	<0.001
BL	4.34 ± 2.28	5.67 ± 2.87	<0.001	4.43 ± 2.40	6.30 ± 3.13	<0.001
ICM	1.50 ± 0.64	1.94 ± 1.18	<0.001	1.44 ± 0.68	2.28 ± 1.84	<0.001
**Flow measurements**						
Blood flux	89.33 ± 40.1	97.98 ± 42.9	0.11^*^	70.99 ± 33.8	83.46 ± 39.5	0.01^*^
MaxV	19.11 ± 7.0	21.66 ± 5.72	0.002	16.69 ± 5.99	19.13 ± 4.57	0.001

*Adjusted p ≤ 0.0025 is the threshold for significance*.

* *There is no significant difference (p > 0.0025)*.

**Figure 4:**
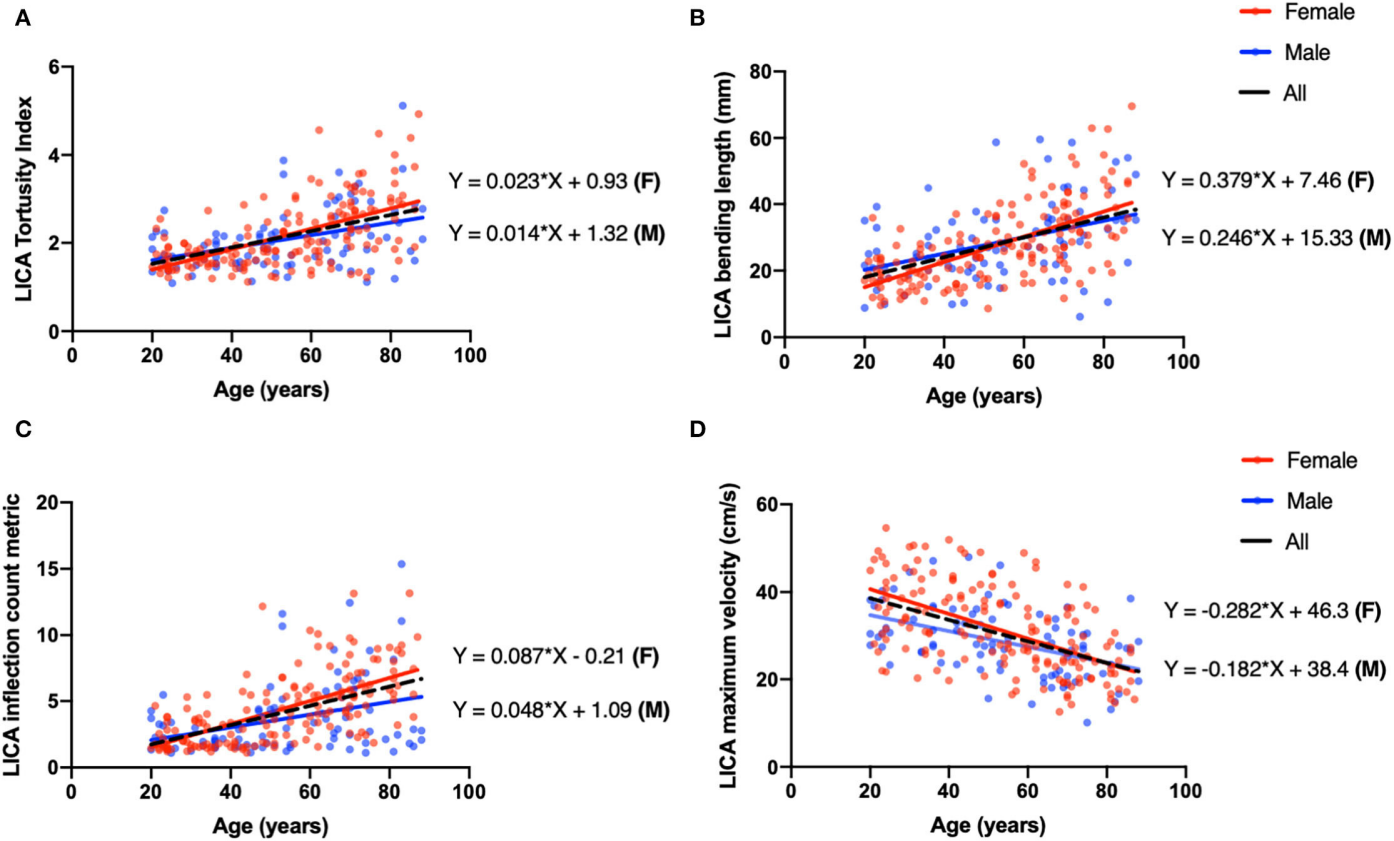
Univariate linear regression plot of left ICA (LICA) as a representative vessel to show the coefficient difference between males (blue) and females (red). **(A)** The TI of LICA has a positive correlation with age (coeff = 0.018, *P* < 0.001, black dash line), and the linear correlation coefficients for males and females are 0.014 and 0.023, respectively. **(B)** Bending length (BL) of LICA is negatively correlated with age (coeff = 0.30, *p* < 0.001, black dash line), and the linear correlation coefficients for males and females are 0.246 and 0.379, respectively. **(C)** The ICM of LICA has significantly positive correlation with age (coeff = 0.072, *p* < 0.001, black dash line). The coefficients for males and females are 0.048 and 0.087. **(D)** The MaxV of LICA is negatively correlated with age (coeff = −0.247, *p* < 0.001, black dash line), and coefficients for males and females are −0.182 and −0.282.

We then applied linear regression model on four vessels to reveal the relationship between age and vascular characteristics in females and males separately. A detailed *r*^2^ and coefficients were summarized in Table 5. Females had higher absolute values of coefficients, reflecting faster changing patterns associated with age, excluding blood flux of ICAs. We then chose LICA as the representative vessel to show the different changing trends of vascular profiles in relation to the age for different genders (Figure 4). Although *t*-test did not reveal significant gender difference for bilateral ICAs tortuosity, the general trend still could be observed that females have relatively higher values at elder stage than males.

**Table 5:** The *r*^2^ and coefficients of univariate linear regression analysis between age and vascular characteristics for males (*n* = 86) and females (*n* = 161).

	**LICA**						**RICA**					
	**Male**			**Female**			**Male**			**Female**		
	***r^**2**^***	***coeff***	***p***	***r^**2**^***	***Coeff***	***p***	***r^**2**^***	***coeff***	***p***	***r^**2**^***	***coeff***	***p***
TI	0.15	0.014	<0.001	0.35	0.023	<1e−4	0.27	0.019	<1e−4	0.28	0.022	<1e−4
BL	0.17	0.24	<0.01	0.40	0.38	<1e−4	0.33	0.33	<1e−4	0.40	0.40	<1e−4
ICM	0.11	0.05	0.002	0.40	0.09	<1e−4	0.19	0.06	<1e−4	0.20	0.08	<1e−4
Blood flux	0.34	−1.75	<1e−4	0.25	−1.66	<1e−4	0.27	−1.51	<1e−4	0.22	−1.47	<1e−4
MaxV	0.21	−0.18	<1e−4	0.30	−0.28	<1e−4	0.14	−0.15	<0.001	0.30	−0.28	<1e−4
	**LVA**						**RVA**					
TI	6e−3	8e−4	0.47^*^	0.03	2e−3	0.04^*^	4e−3	8e−4	0.55^*^	7e−3	2e−3	0.31^*^
BL	0.02	0.017	0.17^*^	0.02	0.02	0.05^*^	0.04	0.02	0.07^*^	0.05	0.04	0.005^*^
ICM	0.13	0.01	<0.001	0.10	0.02	<1e−4	0.05	7e−3	0.03^*^	0.05	0.02	0.005^*^
Blood flux	0.08	−0.58	<0.001	0.07	−0.60	<0.001	0.05	−0.39	0.03^*^	0.11	−0.65	<1e−4
MaxV	0.14	−0.10	<0.001	0.23	−0.17	<1e−4	0.13	−0.10	<0.001	0.20	−0.14	<1e−4

*r^2^: r squared; coeff: coefficients of linear regression; Adjusted p ≤ 0.0025 is the threshold for significance*.

* *There is no significant difference (p > 0.0025)*.

### Association Between VT and Flow

Multivariate linear regression was performed to predict flow properties using age, gender and each tortuosity measurement as independent predictors. The VT had no significant contribution to the blood flow predictions (*p* > 0.05). Significant regression equations were found for MaxV within bilateral ICAs (*p* < 0.001). We then calculated the correlation coefficients between tortuosity and flow measurements. All tortuosity measurements of ICAs showed significantly negative correlation results. A detailed coefficients with 95% CI, adjusted *r*^2^, and *p-*values are summarized in Table 6.

**Table 6:** The VT measurements in relation to the MaxV within ICAs.

**Tortuosity measurements**	***Coeff*** **(95% CI)**	***Adjusted r*** ^**2**^	***p***
**TI**			
LICA	−3.20 (−4.72 to −1.68)	0.32	<0.001
RICA	−3.60 (−5.09 to −2.11)	0.31	<0.001
**BL**			
LICA	−0.21 (−0.31 to −0.11)	0.32	<0.001
RICA	−0.16 (−0.27 to −0.05)	0.27	<0.001
**ICM**			
LICA	−0.69 (−1.18 to −0.35)	0.31	<0.001
RICA	−0.60 (−0.94 to −0.26)	0.28	<0.001

*Coeff, coefficients of linear regression; Adjusted p ≤ 0.0025 is the threshold for significance*.

^*^There is no significant difference (p > 0.0025).

### Accuracy and Reproducibility Evaluation

Regarding the accuracy evaluation in the large dataset of 247 subjects, the algorithm successfully extracted vascular skeleton for 204 subjects of all four arteries, with a subject-based successful rate of 82.6%. Artery-wise, the algorithm successfully provided the vascular skeleton for 941 arteries out of a total of 988 (i.e., 247 × 4) arteries with a success rate of 95.2%. Further investigation showed that we can get satisfied arterial segmentation if appropriate threshold was chosen using multi-level algorithms. However, in cases that the centerline tracking algorithm was failed are likely due to the following reasons: (1) The artery has complicated geometry such as coiling where two segments overlapped with each other; (2) the target vessel (i.e., ICA) was very close to the external carotid artery (ECA) and the algorithm falsely identified the ECA as part of ICA. The aforementioned failed vascular tracking can be improved by increasing the signal threshold so that the signals from unwanted vessels can be excluded and the overlapping between two segments or two vessels can be eliminated.

Among these seven subjects with test–retest scans, the algorithm successfully provided the vascular skeleton for all arteries with no failure. The mean and standard error of inter-session CoV for TI, BL, and ICM measurements were 3.0% ± 0.76%, 7.70% ± 2.27%, and 3.0% ± 0.76%, respectively, across all 28 arteries.

The details of the reproducibility of PC–MRI blood flow quantification have been reported by Liu and Peng et al. (25–27). It has been reported that the inter-session CoV for the blood flux across all 28 arteries was 5.25 ± 2.93% and the inter-session CoV for the unit CBF was reported as 7.41 ± 2.99%.

## Discussion

We applied MRA techniques to neck major brain feeding arteries to reveal the VT and flow changes in a large cohort across the adult lifespan. Older subjects demonstrated higher values of tortuosity measurements. Tortuosity of bilateral VAs are less evident than ICAs, which might be due to the anatomical restriction from the transverse foramina of the cervical spine. Age-related higher VT measurements correlate with lower blood flux and velocity in extracranial arteries, suggesting tortuosity changes in the elderly may limit the efficient blood supply and influence neuronal functions, particularly when neural activity is increased with high energy demand. Further, the results in this study provide quantitative VT measurements in each decade, which can be used as reference values for future clinical studies of patients.

Previous studies suggested that the hypertension was associated with damage of arterial walls, which potentially lead to vessel morphological alterations (6, 28). In this study, we did not find the significant effect of hypertension on VT or flow measurements in our cohort. This discrepancy might be attributable to the following factors. First, the pathophysiological effects of hypertension are dependent on the size of vessels where the resistance and BP will be higher for small vessels with narrow lumen (29). The vessels that, when relaxed, measured lesser than 400 μm in lumen diameter are the major site of vascular resistance, which included a network of small arteries (lumen ≈ 100 to 400 μm) and arterioles (<100 μm). The ICAs and VAs, however, are large arteries with relatively low resistance, and this may be why the tortuosity measurements were less affected by the hypertension. Second, the participants in this normal lifespan study are all healthy subjects from DLBS cohort without history of neurological disorders, such as stroke, transient ischemic attack (TIA), cognitive disorders or MMSE lower than 26. Those pathological conditions potentially complicated the interactions between blood pressure, blood flow, and vasculature topology. Third, the number of hypertensive subjects is relatively small, only 13% had diagnosed hypertension whose symptoms were controlled by taking antihypertensive medication; some of them had normal BP at the time of MRI. They are expected to be part of the normal aging process. Last, the mechanisms of vessel wall remodeling from elevated pulse wave velocity associated with hypertension may be different from that of normal aging. Hypertensive-related changes in large elastic arteries, i.e., ICA, represent as thickening of the vascular wall and increased intima-media thickness (IMT) instead of elastin fracture and collagen deposition seen in normal aging (9, 30). Finally, few studies applied quantitative VT assessment method as used in this study; and most of the previous studies revealed the effects of hypertension purely based on categorical data. However, more studies with larger and chronic hypertensive population are necessary to better understand how hypertension affects the vascular topology.

Gender is another important factor affecting vascular properties. It has been suggested that females are prone to have VT, stiffer large arteries and higher pulse pressure (31–33). While our study did not observe significant difference between females and males in bilateral ICAs, more evident age-related changing patterns can be observed in females. Underlying mechanisms may include variations in sex steroid levels and less stable intervertebral joints in females (34). After menopause, females are prone to stiffer and more tortuous large arteries, resulting from declining female sex steroids secretion associated with decreased elastin/collagen ratios (35). Consequently, the arterial wall remodeling can alter the profile of blood flow.

We hypothesized that vessel morphological changes (e.g., twists and turns) may cause a reduction of arterial pressure and flow velocity in the vessel. Therefore, we analyzed the relationship between VT changes characterized on TOF-MRI and blood flow characterized on PC-MRI. Tortuosity measurements were negatively correlated with blood velocity after adjusting age and gender as covariance, whereas, no association was observed between VT and blood flow. It suggested that age contributes the most to the flow reduction instead of tortuous change based on the multiple regression model. We speculated it was sufficient for the vascular elasticity to regulate the vascular resistance induced by VT aiming to maintain sufficient blood flow under resting conditions (36), whereas this regulatory capacity decreases with aging. The reduced blood velocity is attributable to an elevated resistance and reduced pulse pressure induced by VT. The alteration of flow velocity consequently affects WSS, an indicator of atherogenesis and cognitive function impairment (37, 38), which is determined by the velocity gradient from the vessel wall toward the vessel center. The WSS reduction induced by VT increases the risk of plaque formation mediated by inflammatory factors (39). Several *in vitro* numerical simulation studies have validated that VT results in pressure and flow drops in the distal vessel segments regardless of the bending angles (40, 41). These consequences could further lead to intracranial hypoperfusion and ischemic status.

Extracranial VT changes were investigated extensively with different imaging modalities and our findings are in agreement with most reports, which are largely based on qualitative visual inspection (8, 42–44). There is still an unmet clinical need for quantitative assessment of major brain feeding arteries using clinical imaging. Unlike the most geometric data derived from 2D planar analysis (45–47), our study established an automatic algorithm for 3D vessel segmentation, centerline tracking and VT quantification. While there are several advanced segmentation algorithms developed for complicated vasculature, we applied simplicity multilevel threshold-based segmentation by taking advantage of its fast process and high accuracy (26). Averaged processing time of segmentation, centerline tracking combined with VT computation for a single vessel is within 2 min. In this study, we characterized VT objectively using three rational indices, which addressed the urgency to overcome subjective visual assessment and establish a quantitative tortuosity measurement scheme in clinical settings.

Since our work is built upon a comprehensive quantitative investigation of a large cohort of healthy populations using MRI, the results can potentially serve as normal reference values of each decade in healthy aging. While many studies have been conducted using ultrasound, the accuracy might be limited due to ultrasound's inability and inefficiency to detect thin vessels deep in the tissue or partially hidden behind the bone structures, such as VAs. Meanwhile, the operative-dependent variabilities including the angle of insonation and anatomic location might be introduced. The advantages of MRI over ultrasound include larger coverage, deep vessel detection, and smaller measurement variability (48). Owing to the clinical accessibility and fast acquisition (<5 min), it is practical to perform TOF and PC-MRI as routine scans for quick evaluation of neck vessels regarding intracranial health. The fast and user-interaction algorithm developed in this study makes it feasible to be implemented in MRI scan workstation in the future and provide real-time quantitative measurements of vascular tortuosity.

Some potential limitations of our study should be acknowledged. First, we do not have clinical data regarding some cardiovascular risk factors such as family history of vascular disease, smoking, alcohol use, and hyperlipidemia. Although these risk factors may also be associated with the vessel tortuosity measurements, it is worth noting that the participants in this DLBS study were healthier and more educated than the general population by pre-study screening. Therefore, they were considered as “best-case-scenario” model representing the normal aging effect in the general population with less unwanted noise that potentially confound the biological process (7). Second, in this cohort, there were more female than male participants with the gender ratio almost 2:1, which is primarily due to the reason that females are more willing to participate for this study. However, the detailed tortuosity measurements based on gender were provided in Supplementary Material, and we did not find any gender distribution differences between young and old groups. Third, the vessel diameter or the vessel size was not measured in this study primarily due to the lack of robustness of the techniques used in this study. Last, the tortuosity evaluation for intracranial arteries was not included in this study although morphological variations can present in both intracranial and extracranial arterial segments. This is because the age-related changes of extracranial carotid and vertebral arteries are more common and distinguished due to their eminent anatomical course (no branches) and long length in the neck region. The intracranial segments are often obscured by their complicated route and branches with variable spatial relation with cranial nerve and cavernous sinus (49, 50), which are difficult to be reliably segmented with the methods of this study. The assessment of age-related intracranial VT should be assessed in future studies with specific techniques that are more robust to intracranial segments.

In summary, the levels of age-related VT changes can be quantitatively assessed with the developed vessel segmentation and centerline tracking methods based on fast and non-invasive MRA clinical imaging. Increased tortuosity and decreased blood flow measurements were observed with aging. The blood flow velocity showed an inverse relationship with the tortuosity measurements. The quantitative evaluation of changes of these extracranial major feeding arteries can provide potential indicators of neurological disorders, which would both increase our general understanding of aging and improve clinical treatment and prevention through quantitative analysis of tortuosity levels.

## Data Availability Statement

The original contributions presented in the study are included in the article/Supplementary Material, further inquiries can be directed to the corresponding author.

## Ethics Statement

The studies involving human participants were reviewed and approved by UT Southwestern Institutional Review Board. The patients/participants provided their written informed consent to participate in this study.

## Author Contributions

YG and HL contributed to the conceptualization and design of the study. ZS, DJ, PL, and YC contributed to the methodology and algorithms. ZS, DJ, and PL organized the database. ZS performed the statistical analysis. ZS and YG wrote the first draft of the manuscript. All authors contributed to manuscript revision, read, and approved the submitted version.

## Funding

This study was funded by the National Institutes of Health Grants (RF1 NS11041, R56 AG060822, R01 NS108491, R13 AG067684, P30 AG066512, and P01 AG060882). This study is also supported by Alzheimer's Association (AARG-17-533484).

## Conflict of Interest

The authors declare that the research was conducted in the absence of any commercial or financial relationships that could be construed as a potential conflict of interest.

## Publisher's Note

All claims expressed in this article are solely those of the authors and do not necessarily represent those of their affiliated organizations, or those of the publisher, the editors and the reviewers. Any product that may be evaluated in this article, or claim that may be made by its manufacturer, is not guaranteed or endorsed by the publisher.
