# Supplementary material for: Age-Related Tortuosity of Carotid and Vertebral Arteries: Quantitative Evaluation With MR Angiography
Source: Front Neurol. 2022 Apr 29;13:858805. doi: 10.3389/fneur.2022.858805 (PMC9099009; doi:10.3389/fneur.2022.858805)
Supplement: Supplementary file 1 [file Table_1.PDF]

Supplementary Material

1 SUPPLEMENTARY TABLES AND FIGURES

1.1 Tables

Supplementary Table 1. ICA Vascular tortuosity and flow measurements of each age decade in females.

**Age group**		**20-30 (n = 28)**	**31-40 (n = 22)**	**41-50 (n = 21)**	**51-60 (n = 24)**	**61-70 (n = 29)**	**71-80 (n = 22)**	**> 80 (n = 15)**
Age		24.86±2.97	34.77±3.02	45.33±3.02	55.71±3.16	66.0±3.42	74.0±2.76	83.13±2.23
BP		107/75	106/74	117/80	125/83	135/82	138/85	141/83
TI	LICA	1.66±0.33	1.68±0.3	1.83±0.5	2.08±0.59	2.41±0.71	2.71±0.75	3.04±0.98
	RICA	1.82±0.44	1.85±0.54	2.0±0.63	2.31±0.59	2.75±0.99	2.60±0.62	3.12±0.72
BL	LICA	18.85±6.3	19.76±4.8	21.69±6.49	28.92±9.59	30.93±9.54	37.44±12.01	40.35±13.83
	RICA	19.34±6.67	22.47±7.28	23.84±4.27	32.72±10.31	36.11±14.22	33.24±7.45	45.03±11.5
ICM	LICA	2.27±1.16	2.57±1.14	3.49±2.66	4.55±2.2	5.64±2.21	6.15±2.72	7.23±2.55
	RICA	3.18±2.41	3.27±1.75	3.84±3.13	5.09±2.85	6.61±4.0	7.01±3.23	6.66±3.26
Blood flux	LICA	292.27±55.71	266.11±50.25	247.5±58.22	249.18±63.81	232.88±59.09	201.76±67.72	193.76±44.41
	RICA	285.01±56.43	265.41±46.34	261.52±54.49	226.66±56.45	223.22±64.50	208.37±58.51	210.27±44.18
MaxV	LICA	37.43±8.88	36.04±10.06	37.26±7.86	31.31±8.91	26.85±8.66	26.03±7.11	21.25±5.21
	RICA	38.35±8.73	38.55±7.88	38.98±9.69	32.06±9.29	27.32±8.91	26.67±6.46	25.28±5.26

BP, blood pressure presenting as mean systolic/ mean diastolic. *LICA*, left internal carotid artery; *RICA*, right carotid artery; *TI*, tortuosity index; *BL*, bending length (mm); *ICM*, inflection count metric; Blood flux in ml/min; *MaxV*, maximum blood velocity, in cm/s.

Supplementary Table 2. ICA Vascular tortuosity and flow measurements of each age decade in males.

Age group		20-30 (n = 14)	31-40 (n = 10)	41-50 (n = 15)	51-60 (n = 10)	61-70 (n = 17)	71-80 (n = 10)	> 80 (n = 10)
Age		23.21±2.75	35.10±2.07	45.93±3.08	53.0±1.56	66.65±2.69	74.0±2.36	84.3±2.67
BP		118/ 75	122/83	122/80	131/85	138/87	138/81	141/79
TI	LICA	1.74±0.46	1.81±0.28	1.81±0.35	2.12±0.94	2.27±0.66	2.35±0.78	2.63±1.12
	RICA	1.57±0.35	1.98±0.51	1.83±0.42	1.94±0.62	2.43±0.94	2.52±0.59	2.84±0.91
BL	LICA	21.55±9.3	26.18±8.36	23.85±8.24	27.29±12.65	32.31±11.93	33.65±15.21	37.06±13.65
	RICA	19.77±9.06	29.08±8.12	24.54±8.96	26.85±10.07	31.93±10.2	39.01±9.73	41.58±9.33
ICM	LICA	2.57±1.34	1.96±0.45	2.74±1.48	4.50±3.76	4.59±2.86	4.72±2.66	4.93±4.88
	RICA	1.84±0.88	2.96±2.21	3.09±1.54	4.09±2.65	5.17±3.78	4.57±2.84	6.72±5.35
Blood flux	LICA	274.56±65.9	278.25±36.24	244.52±43.5	236.39±34.07	205.36±47.33	183.36±50.85	180.27±60.94
	RICA	256.27±54.05	265.98±58.72	255.18±49.75	213.82±58.75	196.84±48.6	184.33±24.72	182.24±44.14
MaxV	LICA	33.63±6.15	31.72±7.01	31.13±7.81	31.12±7.46	24.35±6.84	24.45±6.57	23.66±7.72
	RICA	33.12±5.78	30.98±5.79	34.75±7.0	32.55±10.24	25.95±9.63	25.10±4.35	25.06±8.51

BP, blood pressure presenting as mean systolic/ mean diastolic. *LICA*, left internal carotid artery; *RICA*, right carotid artery; *TI*, tortuosity index; *BL*, bending length (mm); *ICM*, inflection count metric; Blood flux in ml/min; *MaxV*, maximum blood velocity, in cm/s.
